# Hospital hand hygiene opportunities: where and when (HOW2)?

**DOI:** 10.1186/1753-6561-5-S6-P112

**Published:** 2011-06-29

**Authors:** CJ Steed, JW Kelly, D Blackhurst, S Boeker, P Alper, E Larson

**Affiliations:** 1 Infection Prevention and Control, Greenville SC, USA; 2 Greenville Hospital System University Medical Cent, Greenville SC, USA; 3 Quality Management, 2Greenville Hospital System University Medical Cent, Greenville SC, USA; 4 Research and Development, Deb Worldwide Healthcare, Inc, Stanley, NC, USA; 5 School of Nursing, Columbia University, NewYork, USA

## Introduction / objectives

The purpose of this study was to estimate hand hygiene opportunities (HHOs) in two types of hospitals -- large-teaching and small-community, and three different clinical areas -- medical-surgical intensive care, general medical wards, and emergency departments.

## Methods

Hand hygiene opportunity data were collected through direct observations using the World Health Organization’s (WHO) monitoring methodology. Estimates of HHOs were developed for 12-hour AM/PM shifts and 24-hour time frames.

## Results

During 436.7 hours of observation, 6640 HHOs were identified. Estimates of HHOs ranged from 30 to 179 per patient day on inpatient wards and 1.84 to 5.03 per bed hour in emergency departments. Significant differences in HHOs were found between the two hospital types and between the three clinical areas.

## Conclusion

This study is the first to use WHO data collection methodology to estimate HHO in general medical wards and emergency departments. These data can be used as denominator estimates to calculate hand hygiene compliance rates when product utilization data are available.

## Disclosure of interest

C. Steed Grant/Research support from DebWorldwide, J. Kelly Grant/Research support from DebWorldwide, D. Blackhurst Grant/Research support from DebWorldwide, S. Boeker Employee of DebWorldwide, P. Alper: None declared, E. Larson Grant/Research support from DebWorldwide.

